# Evaluating Educational Credentials of Teachers as Predictor of Effective Teaching: A Pupil Fixed-Effect Modeling Approach

**DOI:** 10.3389/fpsyg.2021.729360

**Published:** 2021-10-26

**Authors:** Ji Liu

**Affiliations:** Faculty of Education, Shaanxi Normal University, Xi'an, China

**Keywords:** China, learning gains, effective teachers, pupil fixed-effect, educational success

## Abstract

Many factors serve as predictors of effective teaching; particularly, there is an ongoing debate regarding whether the educational credentials of teachers are indicative of their contribution to pupil's educational success. Utilizing pupil fixed-effect modeling and the China Education Panel Survey dataset (*n* = 5,032), this study evaluates the extent to which teachers who hold at least a Bachelor of Education (BEd) degree perform better than those who do not, in terms of pupil learning gains over the course of a full academic year. Empirical results from the pupil fixed-effect model indicate that mean learning gain is 0.042 SDs (95% CI: 0.008–0.083, *p* = 0.040) higher among pupils who studied with teachers holding higher educational credentials (at least a BEd degree) than those with lower educational credentials. This effect translates to approximately 1 month of additional learning per year, which is significant considering the potential compounding aggregation effects over the course of the entire educational career of pupils. This study adds new evidence that highlights the importance of the educational credentials of teachers as a predictor of effective teaching and that better-educated teachers can lead to improved pupil learning gains.

## Introduction

In recent decades, the quality of education has become a key topic of concern among parents, educators, and policymakers. The ongoing economic, social, and information integration holds strong implications for how the prospect of the future is changing, in that globalization, automation, and knowledge economy are driving a broad emphasis on investment in people. Conceptually, a wide range of inputs fed into the process of learning, such as pupil effort, teacher quality, and school facilities, while instructional and organizational practices are key determinants affecting how efficiently these inputs combine and generate meaningful learning engagements. To that end, evidence across international contexts underscores the critical importance of effective teaching for bolstering educational success, encouraging meaningful interaction, and sustaining lifelong learning (Altinok and Kingdon, 2012; Hanushek and Rivkin, 2012; Chetty et al., 2014). More critically, the weight of the influence of teachers on learning outcomes has been shown to be more deterministic in developing countries, where availability of home educational resources and learning opportunities are scarce (Bau and Das, 2017; Liu and Steiner-Khamsi, 2020). Consequently, policymakers have become interested in evaluating how the educational credentials of teachers can serve as promising predictors of effective teaching and in understanding how such observable indicators of teacher quality affect the learning of children (Liu, 2021).

Nonetheless, the empirical literature on the value of the educational credentials of teachers has long been divided. In one camp, scholars argue that educational credential reflects positively on the level of the academic proficiency of a teacher, in addition to the reflections of sufficient cognitive and non-cognitive skills required to design, execute, and engage in teaching activities (Harris and Sass, 2011). The theoretical basis for this view rests on that the human capital development of teachers precedes in importance than that of their pupils, and that their educational mindset, instructional effectiveness, and professional learning all depend on a foundational level of accumulated skills throughout their own teacher education preparation. As a case in point, Jackson and Bruegmann (2009) summarized the attained level of observable human capital development of teachers, such as degree level, licensure and certification status, and score on licensure examination, into a single index and found positive associations with math and reading achievement of pupils. In broad strokes, teaching is a complex task that requires a well-rounded set of cognitive and non-cognitive abilities to excel and not to mention intensive demands for pedagogical skills and subject knowledge for classroom success. Studies supporting this view have persistently shown that the human capital attainment of teachers strongly predicts on-the-job performance and subsequent pupil learning gains in the classroom (Bastian, 2019; Noell et al., 2019).

Conversely, there is an opposing research cluster that casts doubt on the positive link between the educational credentials of teachers and the academic achievement of pupils. In particular, scholars argue that observable teacher traits are inconsistent at best when used as the predictors of effective teaching (Hanushek and Rivkin, 2006; Chingos and Peterson, 2011). These studies directly put in question the usefulness of existing teacher-education arrangements, as well as making uncertain the effectiveness of educational policy initiatives aimed at improving teacher educational attainment and teacher preparation. Accordingly, such controversial division in this literature strongly motivates rigorous empirical evaluations to assess the value-added of the educational credentials of teachers in learning terms.

To address these aforementioned research contentions, this study elects to focus on the case of China, where swift teacher education reforms and reorganization policies have been taking place in light of public demands for a better quality of education (Zhou and Reed, 2005). A key factor linking the Chinese context to the broader international debate on the usefulness of the educational credentials of teachers as a predictor of effective teaching has been the keen policy interest and emphasis of China in improving teacher educational attainment and the quality of teacher education (Liu and Xie, 2021). Historically, the educational credential requirements of teachers in China varied considerably by instructional level. For instance, primary schools have traditionally set high school diploma, or *shizhuan*, as a basic requirement for the entry of a new teacher, whereas secondary schools often require at least a 2-year associate's degree, or *zhuanke*, to be eligible for employment (Ingersoll, 2007).

In recent years, national motivation to increase the supply of teachers who hold higher levels of educational credentials has been strong. In 1999, the State Council initiated *Action Plan to Revitalize Education in the Twenty-first Century* and widely publicized its *Decisions to Deepen Educational Reform and Improve Quality-Oriented Education in a Holistic Way*, which served as policy guidelines in generating national consensus to improve teacher education (Zhou, 2014). As mandated in the *Ten-Five Teacher Education Reform and Development Outline*, the Ministry of Education (2002) posits that all new teachers in compulsory education should hold at least a bachelor's degree, which led to a sharp increase in the percentage of new teachers who hold bachelor-level educational credentials. In addition, the Ministry of Education initiated Free Teacher Education Program (FTEP) in 2007, recruiting more than 46,000 bachelor-degree teacher candidates in its first cycle (Qian et al., 2020). As a result, the proportion of teachers who hold bachelor's degrees drastically increased within a relatively short time frame. According to the Ministry of Education (2016), the percentage of lower-secondary teachers with at least a Bachelor of Education (BEd) degree expanded quintuple times from 12.4% of the total teaching force in 1999 to 59.4% in 2009, and by 2016, approximately 4 out of 5 lower-secondary teachers have at least a BEd degree.

Despite the rapid movement to bachelorize teachers in China, its benefits realized in pupil learning terms have been unclear. For instance, a cluster of scholars increasingly scrutinize this policy movement to improve the quality of a teacher by “bachelorizing” teachers and question whether attaining a higher level of educational credential contributes to effective teaching (Jin, 2007; Zhou et al., 2011; Hu, 2015). In general, there still exists an open debate in academic circles regarding the effects on learning, resulting from the rapid movement to bachelorize teachers, which echoes the broader international discussion regarding the usefulness of the educational credentials of teachers as a predictor of effective teaching. In particular, little is known empirically about the actual benefits of promoting mass teacher education at the bachelor level (Zhou, 2014). This study, through employing an empirical analysis leveraging pupil fixed-effect modeling, evaluate the extent to which the educational credentials of teachers may serve as a predictor of effective teaching and quantify their contributions to learning.

## Materials and Methods

### Subjects

The research design in this study anchors on two key components, namely, the first being the publicly available longitudinal China Education Panel Survey (CEPS) dataset, and the second is leveraging pupil fixed-effect modeling approach. This study uses CEPS baseline (2013) and follow-up (2014) tracked pupil panel dataset, which is collected through a school-based, multistage, multistrata, and probability proportional-to-size sampling (PPS) design. The CEPS is a nationally representative study that includes a set of five independently administered questionnaires, each of which is distributed to pupils and to their parents, teachers, and principals. The combined panel dataset contains rich information on academic achievement, demographics, educational attitudes, and teacher information. Inclusion of subjects is solely based on the availability of teacher-pupil linked data at both baseline and follow-up waves of CEPS, for which 5,032 subjects in the seventh-grade cohort are included in the analytic sample.

### Measures

As with many existing studies, a common concern with analyzing pupil achievement data is the inclusion as a regressor of the lagged pupil learning outcome, because the variable is likely to be measured with error and because any correlation of learning outcomes over time would make the variable endogenous in the specification (Clotfelter et al., 2007). Consequently, this study specifies the key dependent variable as the gain of a pupil in learning outcomes from one period to the other, termed as “pupil learning gains,” which is calculated as the difference in test performance on the core-subject standardized tests, which is observed between baseline and follow-up waves of CEPS. Unlike previous studies, test score information is directly validated and obtained from educational administrative authorities in the CEPS study. All raw test scores are standardized within each subject to display a mean of 70 points and SD of 10 points. The key explanatory variable, i.e., the educational credentials of teachers, is operationally determined as a binary variable, indicating whether teachers hold at least a BEd degree, or otherwise. A rich set of pupil- and teacher-level variables are analyzed as measures to control for potential confounding influence. Empirical validity and reliability information on core-subject standardized test score measures is presented in the descriptive “Results” section.

### Statistical Analysis

This study utilizes a pupil fixed-effect modeling approach to relate variation in educational credentials among teachers to pupil learning gains that varies by subject-teacher pairs. This empirical analytic approach is often referred to as pupil fixed-effect modeling in the psychometrics and econometrics literature (Metzler and Woessmann, 2012), and its utility rests in addressing endogeneity concerns by eliminating confounding effects of observable and unobservable pupil-level factors that remain invariant among subject-teacher pairs. In addition, the rich panel dataset on pupil and teacher information enables the inclusion of control variable vectors, such as pupil attitude and effort as well as teacher background and experience. Operationally, the empirical pupil fixed-effect model was derived by the following mathematical formula:


$$\begin{array}{l} \left[Y_{ijt}-\;{Y^{{}^{\prime }}}_{ij\left(t-1\right)}\right]=\theta \cdot T_{j\left(t-1\right)}+\;\phi \cdot X_{ij\left(t-1\right)}+\;\delta \cdot C_{j\left(t-1\right)}+\;\mu_{i}+\varepsilon_{ijt} \end{array}$$

where the key dependent variable, $Y_{ijt}-\;{Y^{{}^{\prime }}}_{ij\left(t-1\right)}$, is estimated as the difference in the standardized test performance of pupil *i* for teacher-subject pair *j*, i.e., the observed learning gains between baseline and follow-up surveys. The key explanatory variable, i.e., the educational credentials of teachers, as indicated by *T*_*j*(*t*−1)_ is set to equal 1 if teachers hold at least a BEd degree and set to equal 0 if otherwise. To account for pupil learning input, experience, and attitude, which may vary in relation to teacher-subject pairs, the fully specified model also includes *X*_*ij*(*t*−1)_, which is conceived as a pupil-level vector of control variables, consisting of time-lagged measures of participation in private tutoring, frequency of pupil-teacher interaction, and affinity toward the subject. In the same vein, *C*_*j*(*t*−1)_ represents a teacher-level vector of control variables, which includes teacher background information, such as gender, teaching experience, homeroom teaching status, teacher licensure status, teacher job rank, and receipt of teaching award, which may vary across teacher-subject pairs and confound results. Critically, by including pupil fixed-effects (μ_*i*_), the fully specified model effectively minimizes the potentially confounding influence of pupil-level, teacher-level, and school-level factors that are invariant across subject-teacher pairs.

## Results

### Preliminary Analysis

In Table 1, descriptive statistic information is provided for the final analytic sample. A key feature of the pupil fixed-effect model is that there are as many rows of observation per pupil as there are teacher-subject pair categories. Consequently, the analytic sample size in this study is 15,096 since there are three possible teacher-subject pair categories, namely, Home Language, Numeracy, and Foreign Language.

**Table 1:** Sample descriptive statistics information (*n* = 15,096).

	**Definition and metric**	**Mean**
Key explanatory variable		
Hold at least a BEd	Teacher's educational credential,	
	set to equal 1 if holding at least a BEd, and	0.835
	set to equal 0 if otherwise.	
Teacher-level variables		
Female	Teacher's sex,	
	set to equal 1 if female, and	
	set to equal 0 if otherwise.	0.734
Teaching experience	Teacher's length of teaching experience,	14.669
	self-reported in years	(8.668)
Homeroom teaching	Teacher is responsible as homeroom teacher,	
	set to equal 1 if homeroom teacher, and	0.294
	set to equal 0 if otherwise.	
Hold official licensure	Teacher's official teaching licensure status,	
	set to equal 1 if hold official license, and	0.977
	set to equal 0 if otherwise.	
	Teacher's job rank,	
Hold job rank above level 2	set to equal 1 if ranked above level 2, and	0.840
	set to equal 0 if otherwise.	
Hold at least municipal teaching award	Teacher's teaching award,	0.353
	set to equal 1 if hold at least municipal award, and	
	set to equal 0 if otherwise.	
Pupil-level variables		
Private tutoring	Pupil is enrolled in private tutoring,	
	set to equal 1 if enrolled in private tutoring, and	0.146
	set to equal 0 if otherwise.	
Frequent interaction	Pupil reported frequent interaction with teacher,	
	set to equal 1 if reported frequent interaction, and	0.643
	set to equal 0 if otherwise.	
Subject affinity	Pupil indicated favorable attitude to subject,	
	set to equal 1 if favorable attitude, and	0.896
	set to equal 0 if otherwise.	

*SDs in parentheses where appropriate*.

In more detail, 83.5% of teachers report holding at least a BEd degree, 73.4% of teachers are female, the average teaching experience is 14.669 years (SD = 8.668), and 29.4% of teachers are responsible as homeroom teachers. It is also worth mentioning that teachers in China are subject to official teaching licensure requirements, which are legally required unless teachers are undergoing a probational induction period (Ingersoll, 2007). There are 97.7% of teachers in the analytic sample holding official teaching license. Once teachers pass their probational induction period, they may apply through their local education authority for a series of job ranks, commonly beginning from the progressive order of rankless, level 3, level 2, level 1, senior level 2, and senior level 1 (Ministry of Education, 2014). Prior studies have found teacher job rank, or *zhicheng*, to be positively correlated with length of tenure and teaching performance (Wang and and, 2016). In this study, there are 84.0% of teachers ranked above level 2. According to Chu et al. (2015), teachers must meet rigorous requirements in order to be promoted to the next rank, which includes professional development certification and classroom performance audits, which are aimed to assess the mastery of pedagogy, instructional tools, and classroom management of teachers. Additionally, a teaching award is another indicator of teaching performance and is commonly bestowed by various educational authorities, ranging from national-, provincial-, municipal-, district-, and school levels, often through the form of pedagogical skills and classroom management competitions (Liu, 2019). In this study sample, 35.3% of teachers hold at least the municipal-level teaching award.

**Figure 1:**
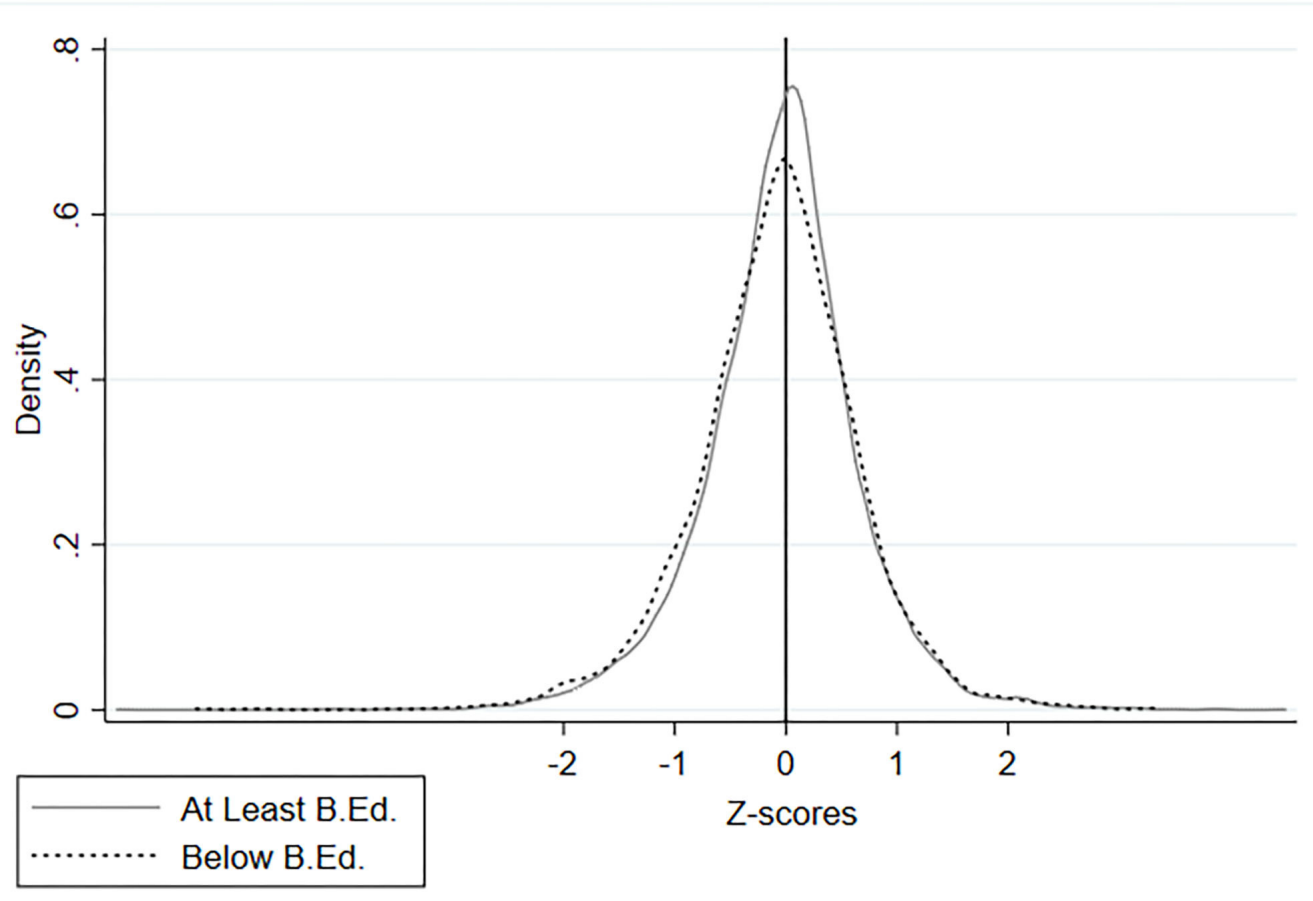
Distribution of pupil learning gains by the educational credentials of teachers. This chart plots the difference in test performance on core-subject standardized tests between baseline and follow-up, by the educational credential of the subject teacher. Kernel density is computed using the Epanechnikov method.

At the pupil level, 14.6% in the sample report as enrolled in private tutoring for Home Language, Numeracy, or Foreign Language, whereas 64.3% feel that their subject teacher frequently interacts with them, and 89.6% indicate that they hold a favorable attitude toward the subject they are learning. Finally, the distribution of the key dependent variable is plotted according to the categories of the key explanatory variable in Figure 1. Visually, it is observed that there is a considerable degree of first-order difference between teachers who hold at least a BEd degree and those who do not, which is depicted in terms of pupil learning gains.

In Table 2, correlation and descriptive statistic information on key dependent variables are presented. Correlational findings confirm that there is good first-order variation among teacher-subject pairs. The correlation coefficients of intersubject test performance at baseline and follow-up, which serve as indicators for reliability among core-subject standardized tests, are relatively high, suggesting that the three tests exhibit reasonable reliability. Specifically, the intersubject correlation coefficients at baseline are 0.641 (*p* < 0.05), 0.720 (*p* < 0.05), 0.70 (*p* < 0.05), for Home Language and Numeracy, Home Language and Foreign Language, as well as Numeracy and Foreign Language, respectively. In addition, the intersubject correlation coefficients at follow-up are 0.69 (*p* < 0.05), 0.68 (*p* < 0.05), 0.73 (*p* < 0.05), respectively, for the same list of subject pairs. For each subject, the intrasubject correlation coefficients between baseline and follow-up, which demonstrate test-retest reliability, are 0.73 (*p* < 0.05), 0.71 (*p* < 0.05), 0.74 (*p* < 0.05), for Home Language, Numeracy, and Foreign Language, respectively.

**Table 2:** Dependent variable correlation matrix and descriptive statistics.

**Dependent variables**	**1**	**2**	**3**	**4**	**5**	**6**		
Baselinetest	1	Home language	1					
	2	Numeracy	0.641^*^	1				
	3	Foreign language	0.720^*^	0.700^*^	1			
Follow-uptest	4	Home language	0.740^*^	0.561^*^	0.631^*^	1		
	5	Numeracy	0.583^*^	0.706^*^	0.612^*^	0.685^*^	1	
	6	Foreign language	0.584^*^	0.615^*^	0.739^*^	0.677^*^	0.732^*^	1
						
*N*	5032	5032	5032	5032	5032	5032		
Mean	75.64	73.73	79.86	78.59	71.53	67.57		
SD	18.22	28.07	26.62	22.06	32.76	30.19		

* *Denotes p < 0.05*.

### Pupil Fixed-Effect Analysis

The main results of the fully specified pupil fixed-effect regression are shown in Table 3, for which the analysis focuses on evaluating the extent to which holding at least a BEd degree serves as a predictor of pupil learning gains between baseline and follow-up. Importantly, the pupil fixed-effect modeling approach limits this analysis within the same pupil, so all confounding influences of pupil-level, teacher-level, and school-level factors that are invariant across subject-teacher pairs are minimized. In more specific terms, the pupil fixed-effect regression coefficient for the key explanatory variable in this study, i.e., “Hold at least a BEd degree,” is evaluated at 0.042 *z*-score units (*p* = 0.040), with a 95% CI between 0.008 and 0.083 *z*-score units.

**Table 3:** Pupil fixed-effect regression results (*n* = 15,096).

**Dependent variable:**				
**Pupil learning gains (*z*-scores)**	**Coefficient**	**SE**	**95% CI**	***p*** **-value**
Key explanatory variable				
Hold at least a BEd degree	0.042^*^	0.021	[0.008, 0.083]	0.040
Pupil-level control variables				
Private tutoring	0.012	0.025	[-0.037, 0.061]	0.639
Frequent interaction	0.015	0.021	[0.026, 0.056]	0.466
Subject affinity	0.082^*^	0.028	[0.027, 0.138]	0.004
Teacher-level control variables				
Female	−0.022	0.017	[-0.055, 0.011]	0.184
Teaching experience	0.002	0.001	[-0.001, 0.004]	0.098
Homeroom teaching	0.058^*^	0.012	[0.034, 0.082]	0.001
Hold official licensure	0.008	0.045	[-0.080, 0.097]	0.856
Hold job rank above level 2	0.065^*^	0.030	[0.007, 0.123]	0.027
Hold at least municipal teaching award	0.001	0.016	[-0.034, 0.031]	0.935

*The adjusted R^2^ of the model is 0.166*.

* *Denotes p < 0.05*.

For interpretation, this finding indicates that pupils who study with a teacher, who holds at least a BEd, are observed on average to exhibit 0.042 more SDs of learning gain between baseline and follow-up than with a teacher who holds less than a BEd degree, holding all else equal, and this result is statistically robust at the 0.05 level. To situate the size of this observed learning gain in a broader context, Evans and Yuan (2019) estimated that pupils are on average predicted to gain between 0.15 and 0.21 SDs of learning per full academic year in developing countries, while the guidance of Organisation for Economic Co-operation Development. (2016) on the number of expected learning gains per full academic year in developed countries is between 0.25 and 0.30 SDs. Conservatively speaking, findings in this analysis suggests that the number of learning that is added by a teacher who holds at least a BEd degree is approximately equivalent to at least 1 month of additional learning per full academic year.

In addition, while the pupil fixed-effect modeling approach accounts for confounding influences that may be invariant across teacher-subject pairs, there may still remain subject-varying pupil-level confounders, such as pupil attitude and effort, as well as subject-varying teacher-level confounders, such as teacher background and experience. Consequently, the full specification of the pupil fixed-effect model includes additional pupil- and teacher-level control variables. As such, the regression results for these important control variable coefficients are also worth mentioning. At the pupil level, participation in private tutoring (*p* = 0.639) and self-reported frequency of interaction (*p* = 0.466) are not statistically correlated with learning gains, whereas self-reported affinity toward the subject (*p* = 0.004) is positively associated with larger learning gains. This result suggests that the affinity of the pupil toward the subject strongly predicts better learning outcomes, even after accounting for subject-invariant factors. At the teacher level, female (*p* = 0.184), teaching experience (*p* = 0.098), official licensure (*p* = 0.856), and municipal teaching award (*p* = 0.935) are not statistically associated with the dependent variable; however, homeroom teaching (*p* = 0.001) and job rank above level 2 (*p* = 0.027) both positively predict larger learning gains. For interpretation, homeroom teaching and job rank are the two important teacher-level predictors for improved pupil learning gains, in addition to the educational credentials of teachers.

## Discussion and Conclusion

Prior studies have indicated that there exists an open debate as to whether observable teacher background traits, such as the educational credentials of teachers, are predictive of teaching effectiveness (Chingos and Peterson, 2011). On the one hand, studies have argued that the human capital development of teachers is vital in determining how well they translate what they know into what pupils learn, particularly in making instructional experiences meaningful to pupils who may possess wide-ranging learning needs (Darling-Hammond et al., 2005). On the other hand, critics of teacher education have asserted that pre-service teacher education is only useful to the extent that teachers are minimally qualified to be a teacher but may not necessarily translate into effective teaching (Ballou and Podgursky, 2005); therefore, many ponder the policy implications since there is some indication that the higher levels of the educational credentials of teachers do not predict elevated learning gains (Hanushek and Rivkin, 2006). Not to mention that academic skeptics have scrutinized recent policy engagements in China, which promote mass teacher education at the bachelor level (Zhou, 2014; Hu, 2015).

The research objective in this study was to evaluate the extent to which the educational credentials of teachers may serve as a predictor of effective teaching and quantify their contributions to pupil's educational success. In broad strokes, this study is centered on the Chinese educational context and employs a pupil fixed-effect modeling approach to relate differences in the educational credentials of teachers to variations in pupil learning gains all within the same pupil, while eliminating problematic confounders at the pupil-, teacher-, and school levels. In broad terms, the Chinese context provides a useful analytic case where policy engagement with improving teacher educational attainment has been swift and strong. More specifically, analytic findings that are generated using baseline and follow-up CEPS datasets indicate that returns to studying under teachers holding higher levels of educational credential are substantial, as calculated in terms of pupil learning gain. Particularly, pupils who study with teachers who hold at least a BEd degree are observed to gain 0.042 higher SDs of measured learning between baseline and follow-up, compared with when the pupil studies with teachers who do not hold such credentials. This effect translates to as much as 1–2 months of additional learning gain per academic year (Organisation for Economic Co-operation Development., 2016). Considering the potentially compounding effect over time, the number of additional learning gains that better-educated teachers can deliver could be quite significant, particularly if pupils are systematically exposed to better-educated teachers in the course of their learning careers.

Findings in this study add rigorous evidence to a growing body of literature highlighting the criticality of the human capital attainment of teachers through programs of tertiary teacher education preparation for boosting instructional effectiveness and bolstering pupil learning and development (Clotfelter et al., 2007; Winters et al., 2012). Conceptually, focusing on improving teacher education and bettering teacher preparation at the tertiary level, well before teachers arrive in the classroom, is an attractive policy option because existing evidence supports that instructional effectiveness matters for learning outcomes (Allen et al., 2013). At the theoretical level, it is crucial to recognize that the human capital development of teachers is a deterministic factor affecting that of their pupils and that how well teachers are educated reflects how well pupils can be reasonably expected to learn. The importance of the level of human capital development of teachers in meeting the increasingly complex demands of instructional success in the classroom should not be underemphasized. As such, a joint policy emphasis should be placed on improving the quality of teacher education and incentivizing teacher candidates in pursuing higher levels of educational credentials. In practice, however, many countries find it a fiscal and logistical challenge to procure large populations of highly qualified teachers, given that the personnel-related costs are already taking up significant portions of educational budgets (Baumol, 1993; Levin, 2011). To that end, while this study does not directly offer fiscal solutions, its findings critically counter against opponents of teacher education and show that better-educated teachers indeed lead to improved learning gains, which can translate into large growth prospects and societal gains in the long run (Hanushek, 2011).

Additionally, closer attention must be paid to understand the inner workings of the Chinese model of teacher education, when contextualizing and translating findings in this study to a culturally and educationally different circumstance. Most importantly, neither all teacher education programs are of the same quality nor do they offer teacher candidates the same professional preparation to make a meaningful difference in the classroom. For instance, Li (2012) observed that the Chinese model of teacher education has been historically a hybrid system that involves a combination of specialized teacher training colleges and comprehensive research universities, and the model emphasizes core values such as independence, adaptability, and diversity stemming from Confucian epistemology and pragmatism. In addition, studies elsewhere have highlighted the importance of cultural relevance in supporting teacher candidates to self-reflect, critique, and link their unique teacher education experience to effective instructional practices in the classroom (Acquah et al., 2020). Finally, in order for teacher candidates to truly benefit from high-quality teacher education programs, studies have shown that they must invest in themselves through hard work, as indicated through undergraduate course grades and course hours (Kukla-Acevedo, 2009).

Nonetheless, two important limitations in this study are also worth mentioning and can perhaps motivate future research in addressing them. First, it is critical to acknowledge that this study only evaluates the extent to which the educational credentials of teachers may serve as an indication for effective teaching, in that its contribution to pupil learning gains is empirically calculated. There are many additional dimensions of human capital development traits of teachers, which are worth considering, such as teacher certification (Harris and Sass, 2009) and teaching experience (Kane et al., 2008), as well as teacher rank in the Chinese context, where the findings in this study, as well as that in the studies by Hannum and Park (2007) and Chu et al. (2015), have shown to positively influence pupil learning. These positive signals of teacher quality are not only indicative of the human capital development of teachers but also more importantly predict their instructional effectiveness when they enter the classroom. Second, the CEPS study does not include in its research design to randomly assign teachers to pupils, and therefore, there are reasonable concerns that sample selection bias at both the school- and classroom level may be present and confound findings. For instance, if more qualified teachers are systematically assigned to teach classes enrolled with high performers, or if more qualified teachers are compensatorily assigned to teach in low-performing classes, the relationship between the educational credentials of teachers and the pupil learning gains becomes ambiguous. Notwithstanding, the pupil fixed-effect modeling approach attempted to address some of these issues to the possible extent, by leveraging within-pupil, between-subject variation, in addition to including a rich set of pupil- and teacher-level control variables. In this regard, however, the estimated effect of the educational credentials of teachers could reflect a combined effect, to some extent, comprised of unobserved peer characteristics at both pupil- and teacher levels. Consequently, future research could readily take advantage of the ongoing longitudinal CEPS and build on findings in this study to explore how sample selection bias may be at play.

## Data Availability Statement

China Education Panel Survey dataset is publicly-available at https://doi.org/10.18170/DVN/KURJUU.

## Ethics Statement

Written informed consent to participate in the study was obtained by National Survey Research Center of Renmin University, PR China. All research ethics approvals were granted by the Institutional Review Board of Renmin University, PR China.

## Author Contributions

The author confirms being the sole contributor of this work and has approved it for publication.

## Funding

This study was financially supported by the National Social Science Foundation of China (Grant No. CJA200256).

## Conflict of Interest

The author declares that the research was conducted in the absence of any commercial or financial relationships that could be construed as a potential conflict of interest.

## Publisher's Note

All claims expressed in this article are solely those of the authors and do not necessarily represent those of their affiliated organizations, or those of the publisher, the editors and the reviewers. Any product that may be evaluated in this article, or claim that may be made by its manufacturer, is not guaranteed or endorsed by the publisher.
